# Supplementary material for: Incoherence between Systemic Hemodynamic and Microcirculatory Response to Fluid Challenge in Critically Ill Patients
Source: J Clin Med. 2021 Feb 1;10(3):507. doi: 10.3390/jcm10030507 (PMC7867072; doi:10.3390/jcm10030507)
Supplement: Supplementary file 1 [file jcm-10-00507-s001.pdf]

Additional files

Additional file 1. Proportion of patients responding to fluid challenge according to changes in cardiac index (CI, i.e. > 10%) or to changes in the proportion of perfused small vessels (PPV, > 10%), in relationship to the use of norepinephrine or sedation. Total of fluid challenge = 41.

		**Responders PPV**	**Non-responders PPV**
**Norepinephrine, (n=24)**	**Responders CI**	6	8
	**Non-responders CI**	3	7
**No norepinephrine, (n=17)**	**Responders CI**	5	5
	**Non-responders CI**	3	4
**Sedation, (n=24)**	**Responders CI**	10	6
	**Non-responders CI**	3	5
**No Sedation, (n=17)**	**Responders CI**	3	5
	**Non-responders CI**	3	6

Additional file 2. Correlation analysis in the subgroups of patients treated (right) or not (left) with norepinephrine drug infusion. CI, cardiac index; PPV, proportion of perfused small vessels; MFI, mean flow index.

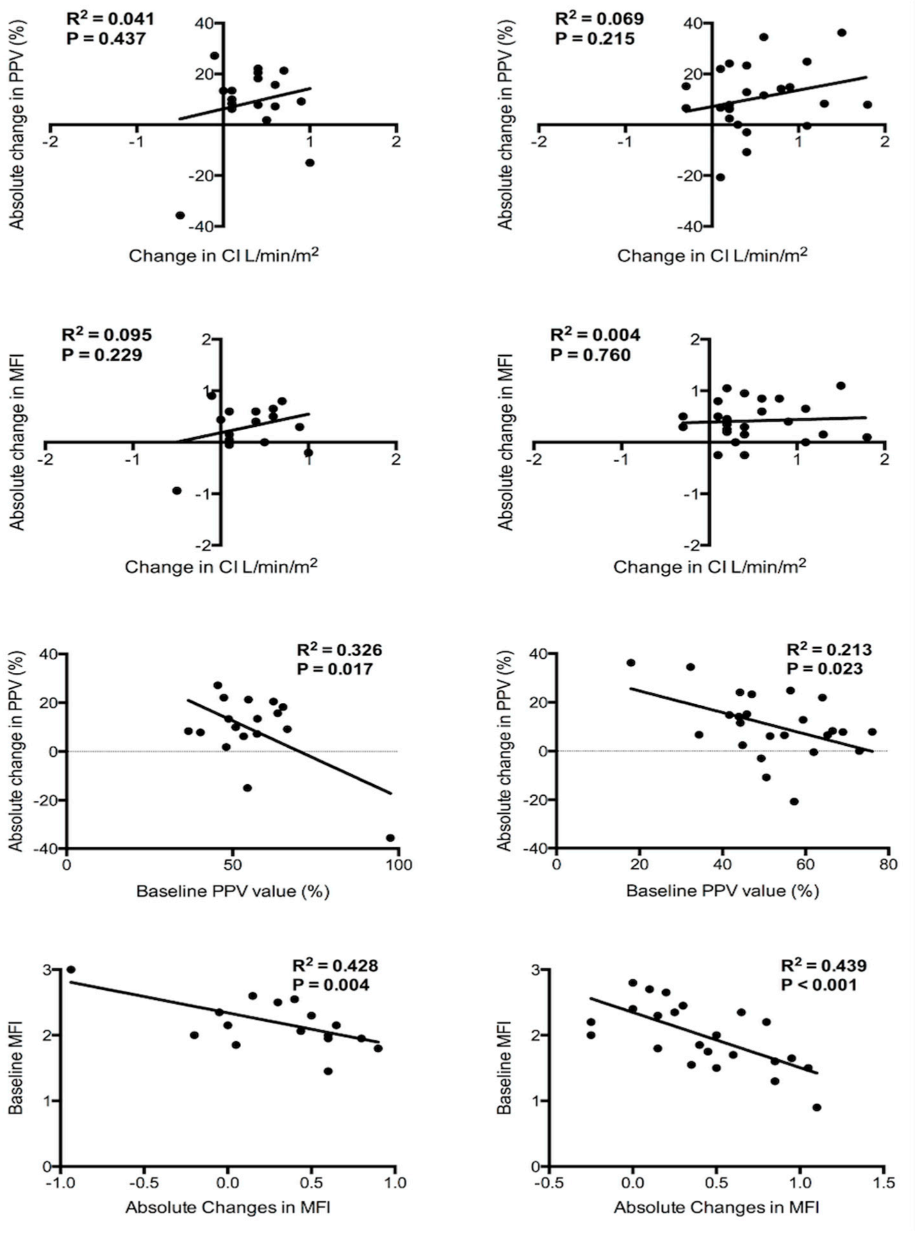

Additional file 3. Correlation analysis in the subgroups of patients treated (right) or not (left) with sedation therapy. CI, cardiac index; PPV, proportion of perfused small vessels; MFI, mean flow index.

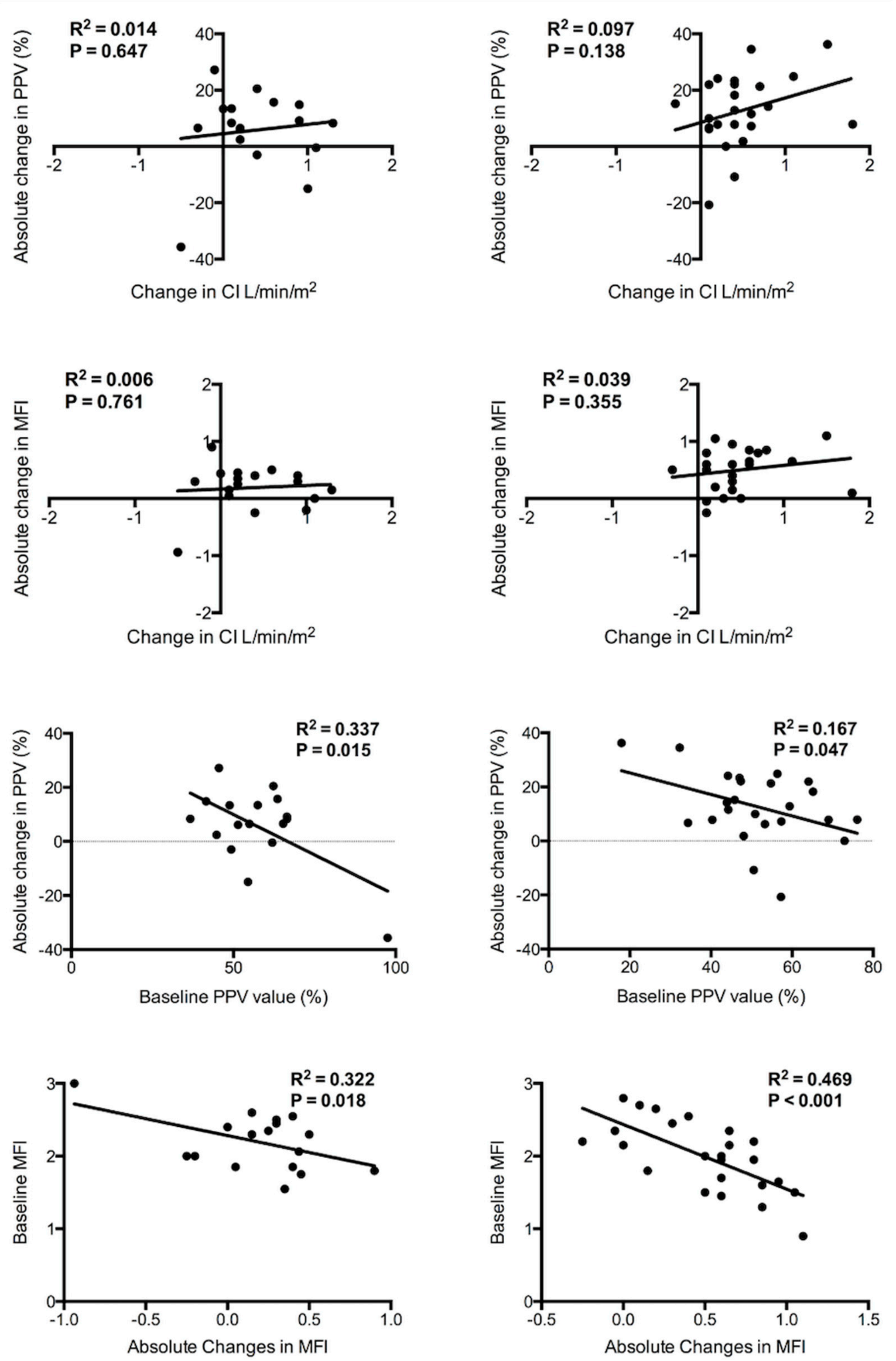

Additional File 4. Univariate analysis between responders and non-responders to fluid challenge according to changes in the proportion of perfused small vessels greater (i.e. > 10%).

Variable	Non Responders N = 22	Responders N = 19	P value
Hemodynamics			
**Heart rate,** beat per minutes	93 [81-100]	85 [74-112]	0.862
**Mean arterial pressure,** mmHg	72 [69-81]	68 [63-74]	0.124
**Central venous pressure,** mmHg	8 [6-11]	7 [6-12]	0.668
**Cardiac index,** L/min/m^2	2.7 [2.3-3.1]	2.6 [2.1-3.1]	0.998
**Lactate,** mmol/L	1.5 [1.0-2.3]	1.8 [1.1-4.1]	0.114
**Central oxygen venous saturation,** %	70 [61-75]	71 [63-81]	0.099
**Venous arterial carbon dioxide gap,** mmHg	8 [6-9]	8 [6-11]	0.421
Microcirculation			
**Total vessel density**, mm/mm2	15.1 [13.2-18.2]	14.6 [12.4-18.3]	0.091
**Density of perfused small vessel**, mm/mm2	8.7 [6.4-10.3]	6.8 [5.7-10.2]	0.700
**Proportion of perfused small vessels,** (PPV) %	55 [49-66]	47 [44-59]	0.072
**Microvascular flow index,** (MFI)	2.2 [1.8-2.5]	1.9 [1.6-2.3]	0.116
**Heterogeneity of MFI,** (%)	0.44 [0.30-0.61]	0.52 [0.35-0.69]	0.192
**Heterogeneity of PPV,** (%)	0.56 [0.36-0.73]	0.52 [0.35-0.69]	0.178

PPV = proportion of perfused vessels; MFI = mean flow index; fluid challenge = 41.
